# No evidence for cold-adapted life-history traits in cool-climate populations of invasive cane toads (*Rhinella marina*)

**DOI:** 10.1371/journal.pone.0266708

**Published:** 2022-04-07

**Authors:** Uditha Wijethunga, Matthew Greenlees, Melanie Elphick, Richard Shine

**Affiliations:** 1 School of Life and Environmental Sciences, University of Sydney, Sydney, NSW, Australia; 2 School of Natural Sciences, Macquarie University, Sydney, NSW, Australia; University of Iceland, ICELAND

## Abstract

As an invasive organism spreads into a novel environment, it may encounter strong selective pressures to adapt to abiotic and biotic challenges. We examined the effect of water temperature during larval life on rates of survival and growth of the early life-history stages of cane toads (*Rhinella marina*) from two geographic regions (tropical vs. temperate) in the species’ invaded range in eastern Australia. If local adaptation at the southern (cool-climate) invasion front has extended the cold-tolerance of early life-stages, we would expect to see higher viability of southern-population toads under cooler conditions. Our comparisons revealed no such divergence: the effects of water temperature on rates of larval survival and growth, time to metamorphosis, size at metamorphosis and locomotor performance of metamorphs were similar in both sets of populations. In two cases where tropical and temperate-zone populations diverged in responses to temperature, the tropical animals performed better at low to medium temperatures than did conspecifics from cooler regions. Adaptation to low temperatures in the south might be constrained by behavioural shifts (e.g., in reproductive seasonality, spawning-site selection) that allow toads to breed in warmer water even in cool climates, by gene flow from warmer-climate populations, or by phylogenetic conservatism in these traits.

## Introduction

In ectotherms, environmental temperature controls developmental rates and a wide range of physiological processes [1]. Traits such as embryonic developmental trajectory, time to metamorphosis, body size at maturity, locomotor performance and growth rate are sensitive to developmental temperature [2–6]. As a result, geographical variation in thermal environments can limit the distribution of a species. At such a range-edge, a species may be under intense selection to adjust its tolerances to the thermal challenges it encounters, either by physiologically-based adaptation or behavioural flexibility [7, 8]. Phenotypically plastic responses commonly adjust organismal traits to the local environment [9]. Many studies on amphibians have documented geographic variation in traits that plausibly enhance the ability of range-edge individuals to survive under the relatively extreme environmental conditions that they encounter. Those traits include the critical thermal minimum [9–11] and maxima [12], morphology [13], embryonic developmental rates [14] and body sizes [15].

How rapidly can such adjustments occur? Invasive species provide a robust model system with which to examine the time course of initial responses to encountering novel challenges [16–18]. A growing literature on invasive species provides many examples of adaptations that enhance dispersal rates, reduce predation vulnerability, and facilitate competitive superiority against native taxa [19–23]. Cane toads in Australia have been intensively studied in this respect, but most attention has focused on the toad’s invasion of tropical habitats [24, 25]. At their southeastern (cool-climate) range-edge, cane toads are moving slowly through coastal habitats [26–29]. Low temperatures in this region may be limiting range-expansion [29, 30].

Studies in other parts of the toads’ Australian range have revealed rapid evolutionary changes in traits that affect dispersal rate [31, 32], life-history traits [33–35], hydric balance [36, 37] and thermal biology [10, 11, 38]. Given these rapid changes, toads may also have evolved an ability to deal with (or at least, exhibit sufficient plasticity to tolerate) low temperatures at their southern range-edge. To evaluate this possibility, we conducted laboratory studies to measure responses of early life-stages of tropical versus temperate-zone cane toads to a range of water temperatures.

## Results

### Hatching success of eggs

**Fig 1:**
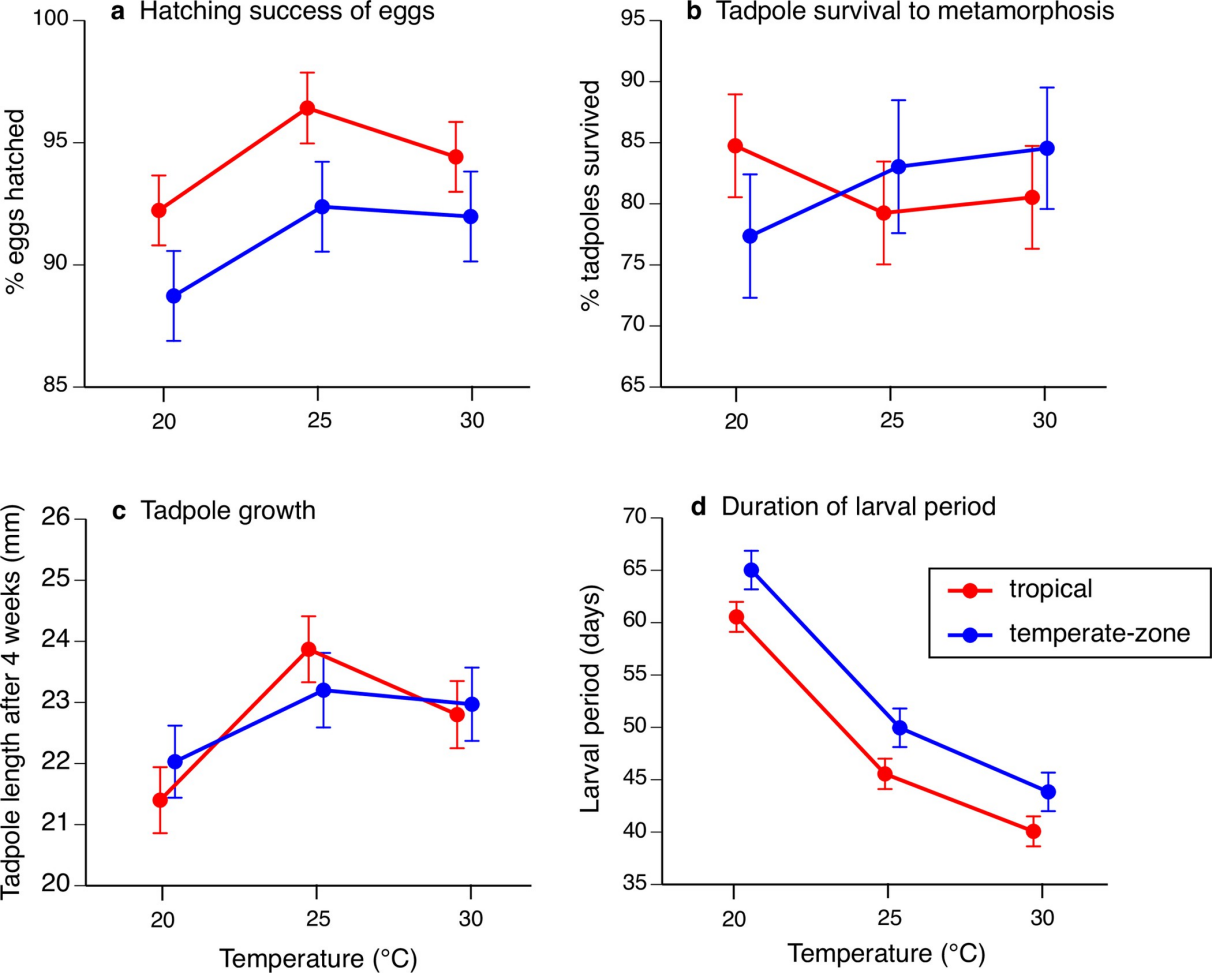
The effects of temperature at which eggs and larvae of cane toads *Rhinella marina* were raised on hatching success, larval survival, larval growth rate and duration of the larval stage. Graphs show effect of rearing temperature on (**a**) hatching success of eggs, (**b**) survival rates of tadpoles from hatching to metamorphosis, (**c**) body lengths of tadpoles at four weeks of age, and **(d)** the overall time taken from hatching until metamorphosis. The panels show data from toads whose parents were collected in two locations: tropical (red) and temperate-zone (blue) Australia (Queensland and New South Wales respectively). The figure shows mean values and associated standard errors based on raw data, although some statistical tests in the text are based on arcsin-transformed or ln-transformed values. Tadpoles from tropical populations had higher hatching success (a) and a shorter duration of larval life (d) at all test temperatures, but rates of survival and growth (b and c) did not differ significantly between tropical vs temperate-zone toads.

The eggs of tropical toads had higher hatching success than did those of temperate-zone toads at all temperatures tested (main effect of location F_1,87_ = 12.76, P < 0.001; interaction temperature*location F_2,87_ = 0.19, P = 0.82; see Fig 1A). Mean hatching success was higher at 25°C than at warmer or cooler temperatures, but this effect did not attain statistical significance (F_2,87_ = 2.80, P = 0.07).

### Rates of survival and growth of tadpoles

Tadpole survival was not significantly affected by the location of origin (F_1,94_ = 0.007, P = 0.93) or by temperature (F_2,94_ = 0.13, P = 0.87; interaction location*temperature F_2,94_ = 0.91, P = 0.40; Fig 1B). Rearing temperature influenced larval growth rate (F_2,100_ = 8.56, P < 0.001) in similar ways in the two populations (location F_1,100_ = 0.009, P = 0.92; interaction location*temperature F_2,100_ = 1.07, P = 0.34). Tadpoles grew more slowly at 20°C than at the other two temperatures (Tukey posthoc 20°C < 25°C or 30°C; see Fig 1C).

### Duration of larval period

Metamorphosis was delayed if tadpoles were kept at lower temperature (F_2,2594_ = 661.91, P < 0.0001; posthoc tests show that all three treatments differ significantly from each other), and was delayed in temperate-zone toads compared to tropical conspecifics (main effect location F_1,2594_ = 86.32, P < 0.0001; interaction location*temperature F_2,2594_ = 0.37, P = 0.69; Fig 1D).

### Mass at metamorphosis

**Fig 2:**
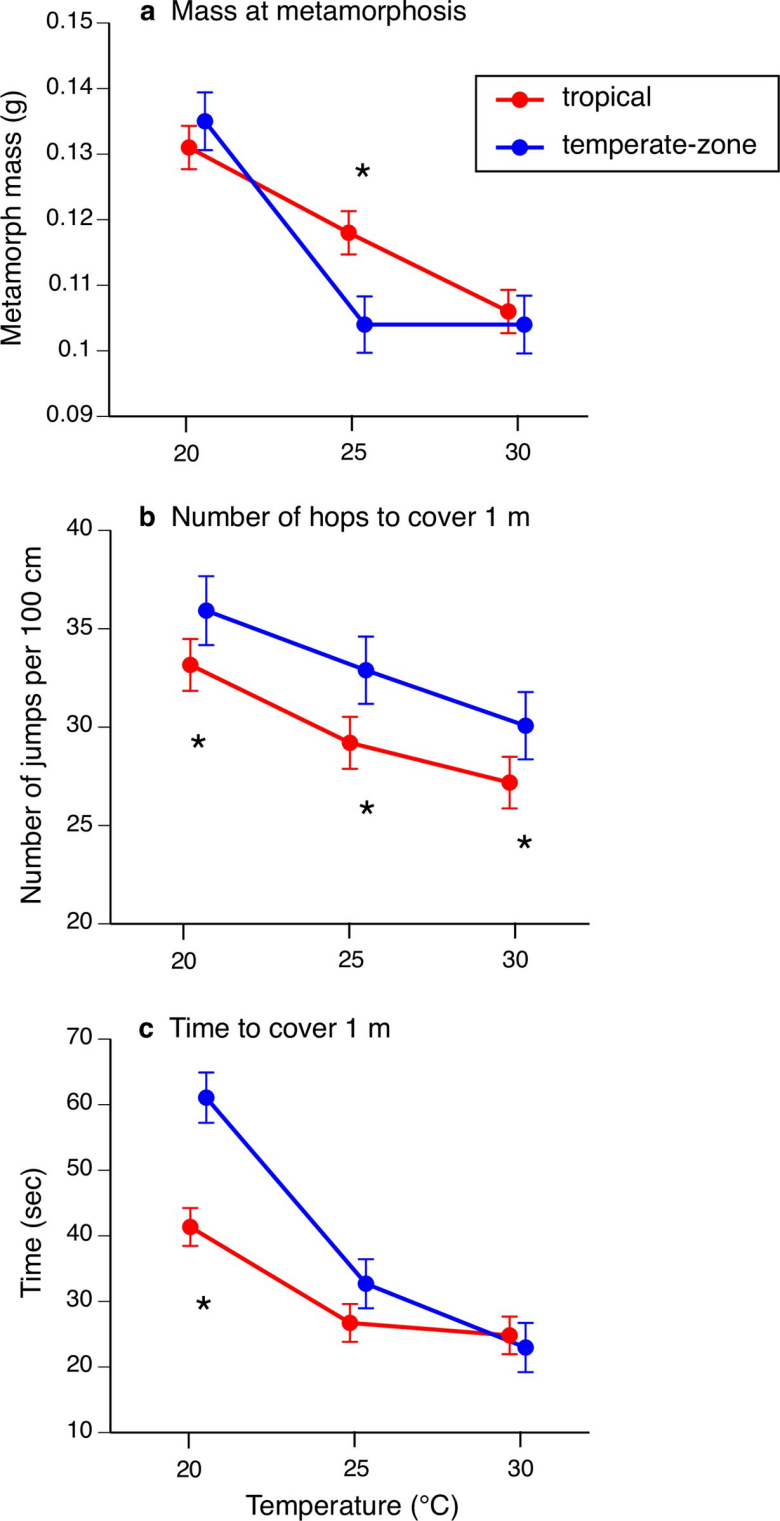
The effects of temperature at which eggs and larvae of cane toads *Rhinella marina* were raised on body size at metamorphosis, locomotor behaviour, and locomotor speed of metamorphs. Graphs show effect of rearing temperature on (**a**) mass at metamorphosis, (**b**) number of hops required for a metamorph to travel 1 m in laboratory raceway trials, and (**c**) the time taken for a metamorph to travel 1 m in laboratory raceway trials. The panels show data from toads whose parents were collected in two locations: tropical (red) and temperate-zone (blue) Australia. The figure shows mean values and associated standard errors based on raw data, although some statistical tests in the text are based on arcsin-transformed or ln-transformed values. Asterisks show location of significant geographic (tropical vs temperate) differences identified by posthoc tests.

Metamorphs from 20°C were larger than were those from warmer temperatures (F_2,2594_ = 127.59, P < 0.0001), with a significant interaction between temperature and location (F_24,2594_ = 12.76, P < 0.0001); a rearing temperature of 25°C resulted in smaller metamorphs in southern temperate-zone New South Wales (NSW) toads than in tropical Queensland (Qld) toads (see Fig 2A).

### Locomotor performance of metamorphs

Tadpoles raised in cooler water produced metamorphs that took shorter hops (i.e., needed more hops to cover the same distance; F_2,1112_ = 39.28, P < 0.001; posthoc tests show that all three treatments differ significantly from each other) and temperate-zone toads took shorter hops than did tropical conspecifics (location effect F_1,1112_ = 47.56, P < 0.0001; interaction temperature*location F_2,1112_ = 0.60, P = 0.55; see Fig 2B). The time required for a metamorph to travel all the way along a 1-m raceway was greater for animals that had been raised in cooler water during larval life (temperature effect F_2,1112_ = 166.24, P < 0.0001), and the increase in time taken after low-temperature rearing was greater for temperate-zone (NSW) metamorphs than for tropical (Qld) metamorphs (interaction temperature*location F_2,1112_ = 18.82, P < 0.0001; see Fig 2C).

## Discussion

Organisms that are expanding their range (like the cane toad in Australia) may encounter novel environmental conditions. They may cope with such challenges either physiologically or behaviourally. Although previous work has reported rapid adaptation and behavioural adjustments of cane toads in response to selective forces within tropical and arid environments [31, 32, 36, 39] and shifts in thermal minimum tolerances in adult toads encountering cool conditions [10, 11], we saw no such shifts in life-history traits of the early life-stages of toads in response to water temperature. Although toads in southern Australia are exposed to waterbodies that are substantially cooler than those used for spawning in tropical Australia [6, 40] or the toads’ native range [41], the thermal responses of eggs and tadpoles were very similar in tropical versus temperate-zone populations. In an analogous result, Volpe [42] found no difference in response to experimental temperature of *Incilius* (*Bufo*) *valliceps* embryos from thermally diverse breeding environments (early and late breeding seasons).

Overall, our results highlight the broad thermal tolerance of *Rhinella marina* eggs and tadpoles. Substantial numbers of eggs hatched, and tadpoles survived and grew, even at 20°C–cooler than in potential spawning ponds at the southern edge of the toad’s current Australian range [6]. Such eurythermy may enable the cane toad to breed successfully in a wide range of conditions, and selection to enhance viability at low temperatures accordingly may be weak. Rearing temperature significantly affected many life-history traits, as previously reported by Wijethunga et al. [6], but we lack field data to assess the fitness consequences of variation in traits such as larval duration and size at metamorphosis.

The only traits for which we detected significant differences between populations in their thermal responses were mass and hopping speeds post-metamorphosis. Temperate-zone toads had smaller metamorphs at an intermediate rearing temperature (25°C) than did their tropical counterparts (Fig 2A). For locomotor speeds, cooler conditions during larval life reduced performance more in temperate-zone toads than in their tropical counterparts (Fig 2B, 2C). Neither of these effects is easily reconciled with the idea of adaptive changes to cool environmental conditions. The durations of larval life were broadly similar in tropical and temperate-zone toads (Fig 1D), so that smaller size at metamorphosis in southern (temperate-zone) animals cannot be interpreted as a tradeoff for faster development [43]. All else being equal, a larger size at metamorphosis is likely to enhance fitness, because smaller metamorphs are more vulnerable to threats such as desiccation, predation, and parasite attack [44]. For locomotor speed, likewise, faster speeds are likely to enhance rather than reduce metamorph fitness; and thus, the slower locomotion of temperate-zone (NSW) metamorphs raised at low temperatures seems unlikely to be adaptive.

The overall similarity in thermal dependency of survival, growth and performance between widely-separated (tropical vs. temperate) populations might reflect a lack of selection for cold-tolerance in eggs and larvae at the southern range edge. For example, the southern limit of the toad’s distribution may be determined primarily by traits such as weather-limited foraging opportunities for adult toads [30] with weak or no selection on larval thermal biology. Selection for cold-tolerance would be further reduced if females delay spawning until summer weather brings pond temperatures close to those experienced year-round in tropical Australia. Local adaptation also may be opposed by gene flow from surrounding populations exposed to higher temperatures [45] or frequent human-assisted translocation of toads to the southern range-edge from warmer climates [46]. Alternatively, toads may not have been exposed to these cool-climate conditions for long enough to adapt [47]. Selection of open sun-exposed ponds [48] may also increase effective water temperatures in the spawning sites used by cane toads in the southern parts of their range. Behavioural plasticity in the times and places used for breeding could reduce or eliminate any selective advantage to low-temperature tolerance of early life-stages [16, 49].

Even if natural selection favours cold tolerance at the expanding range-edge, however, that selection may be ineffective because of tradeoffs with other traits under selection (such as increased dispersal rates at expanding range edges [30–33]). In summary, our results do not provide any support for the prediction that cane toads are evolving cold-tolerant eggs, larvae and metamorphs as the species penetrates southwards into cooler parts of Australia.

## Materials and methods

### Study area and species

Since they were introduced to Queensland (Qld) in 1935, cane toads have spread west across tropical Australia and south along the New South Wales (NSW) coast, sandwiched between mountains to the west and the Pacific Ocean to the east [29, 50]. Cane toads are large bufonids whose powerful toxins kill many native Australian vertebrates [24]. Female cane toads lay up to 30 000 eggs in a string in shallow ephemeral ponds [51]. Previous field studies have reported that in the Australian tropics, cane toads spawn in water averaging 32°C [40]; but conditions are cooler (~28.5°C in summer, 20°C in spring, 24°C in autumn) in their southern temperate-zone breeding ponds [6, 48].

**Fig 3:**
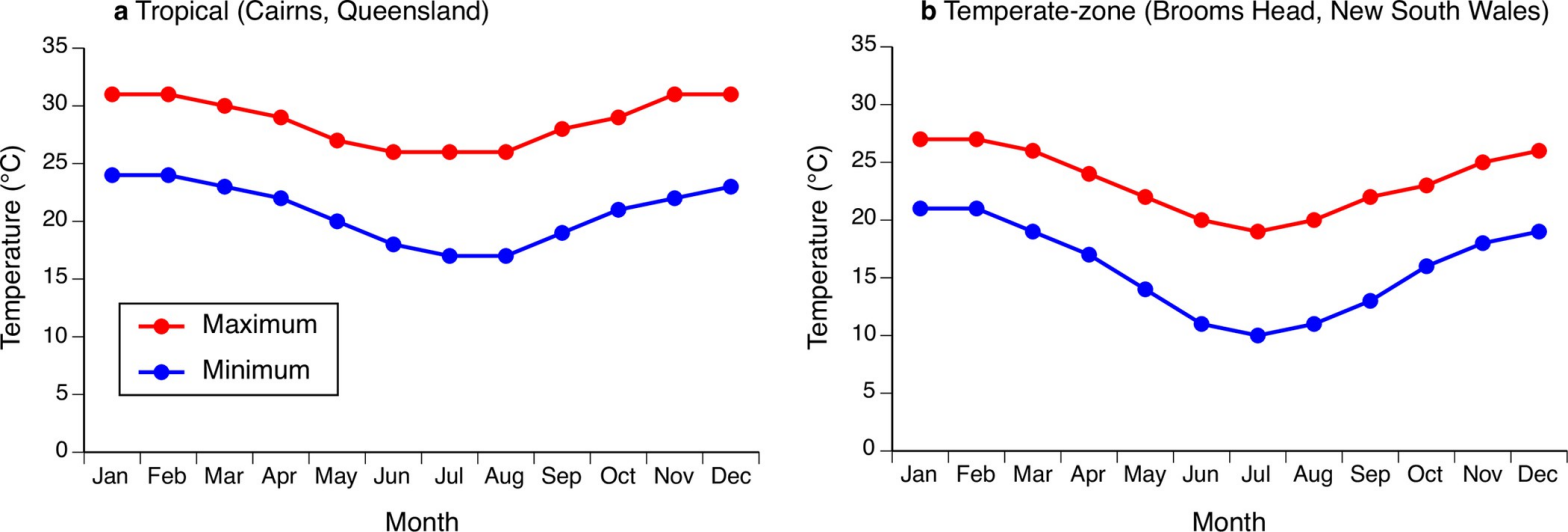
Average monthly air temperatures (maxima and minima) for study sites. (**a**) tropical Australia (Cairns, Queensland), and (**b**) temperate-zone Australia (Brooms Head, New South Wales) from which cane toads (*Rhinella marina*) were collected, to provide offspring tested in the current study. Data from http://www.bom.gov.au (accessed 22 July 2021).

To investigate whether toads from the cool southern (temperate-zone) versus warm northern (tropical) parts of their Australian distribution differ in cold tolerance, we collected adult male and female toads from three populations in tropical Australia (Queensland; centered on 18°00’19.1”S, 146°00’31.1”E, where they have been established for 80 years) and from three populations from the southernmost range-edge in temperate-zone Australia (northeastern New South Wales; centered on 33°56’38.69”S, 147°56’57.41”E) where toads have been present for less than 20 years [29]. Climatic conditions are much warmer at the tropical site than at the temperate-zone site (see Fig 3 for data on mean monthly air temperatures).

## Methods

Adult toads were housed at the University of Sydney for one month, in tubs (390 x 280 x 140 mm; two toads per tub) with ad lib access to water, and food provided three times a week. Room temperature was 22–23°C. The adult toads were then injected with hormone (0.5 mL/L Lucrin, Abbott Australasia, Botany, NSW; see Kelehear et al. [52] for details) in an attempt to obtain two clutches of eggs from each population (i.e., six clutches per site). In practice, we obtained five viable clutches from tropical Queensland toads (two each from Townsville and Tully, one from Cairns) and three from temperate-zone areas in New South Wales (two from Bungawalbin, one from Brooms Head). Strands of 100 eggs from each clutch were then raised at 20, 25, or 30°C, bracketing the range of temperatures available at the tropical (Qld) and temperate-zone (NSW) breeding ponds in Australia. Each temperature treatment contained four replicates, and was maintained using aquarium heaters (AquaWorld, Model HT 2150, Boyu Group, Guangdong, China), with temperature recorded daily (EC-PCST Testr35 multiparameter, model PTTEST35, Eutech, Singapore; accuracy ±0.01°C). The water was aerated for 15 min each day with a 220–240-V aerator (Resun LP-100, Longgang, Shenzhen, China). Tadpoles were fed with frozen lettuce every day, with weekly water changes. Water within each tub (28 x 38 x 20 cm) was conditioned using tap water conditioner (API Tap Water Conditioner, Chalfont, Pasadena; 5 mL per 20 L). We recorded egg hatching success (% hatched), and % survival and body lengths of tadpoles after four weeks.

When tadpoles metamorphosed (completely resorbed their tails), we recorded the date and weighed them. Metamorphs were raised in tubs (390 x 280 x 140 mm; half water, half sand), with each tub containing 20 metamorphs from the same thermal treatment. Food (day-old crickets) was provided three times per week. Room temperature was 22–23°C. The young toads were fed with one-day-old crickets twice weekly. Locomotor performance of metamorphs was tested at 4 weeks of age by placing each animal at the start of a 1-m-long raceway and prompting it to run by gently taping the urostyle with a soft brush. Room temperature was held constant (23–24°C) and we recorded the total time taken to travel 1 m, and the number of hops required.

### Ethics statement

All experimental protocols were approved by the University of Sydney Animal Care and Ethics Committee (Animal Care and Ethics Protocol Number L04/08-2012/3/5807) and were conducted in accordance with all relevant guidelines and regulations, including the ARRIVE guidelines. Euthanasia was performed humanely by immersion in MS-222. Husbandry methods were designed to assure that animals were maintained in good health and without stress.

### Statistical analysis

Analysis of Variance (ANOVA) was performed to compare northern tropical (Qld) versus southern temperate-zone (NSW) populations of toads in terms of % of eggs hatching, % tadpole survival, length of the tadpoles after 4 weeks, time to metamorphosis, mass at metamorphosis, and locomotor ability (time and number of hops required to travel 1 m). To improve normality of distributions, we arcsin-transformed data on the proportion of eggs hatching and proportion of tadpoles surviving, and ln-transformed data on metamorph mass and locomotor traits (the number of hops and times taken required to cover 1 m on the raceway). The independent variables were location (tropical/temperate-zone) and temperature of rearing treatment. We also included clutch # as a random variable, and replicates within clutch as a nested effect (JMP version 15: SAS Institute lnc., Cary, NC). Preliminary analyses revealed no significant link between performance and body size, so we did not include mass as a covariate in the analyses of locomotor traits.

## References

[1] Smith-Gill SJ, Berven KA (1979) Predicting amphibian metamorphosis. Am Nat 113: 563–585.

[2] Harkey GA, Semlitsch RD (1988) Effects of temperature on growth, development, and color polymorphism in the ornate chorus frog *Pseudacris ornata*. Copeia 1988: 1001–1007.

[3] Alvarez D, Nicieza A (2002) Effects of temperature and food quality on anuran larval growth and metamorphosis. Funct Ecol 16: 640–648.

[4] Watkins TB, Vraspir J (2006) Both incubation temperature and posthatching temperature affect swimming performance and morphology of wood frog tadpoles (*Rana sylvatica*). Physiol Biochem Zool 79: 140–149. doi: 10.1086/498182 16380935

[5] Smith GD et al (2015) Effects of temperature on embryonic and early larval growth and development in the rough-skinned newt (*Taricha granulosa*). J Therm Biol 51: 89–95. doi: 10.1016/j.jtherbio.2015.03.010 25965021

[6] Wijethunga U, Greenlees M, Shine R (2016) Moving south: effects of water temperatures on the larval development of invasive cane toads (*Rhinella marina*) in cool-temperate Australia. Ecol Evol 6: 6993–7003. doi: 10.1002/ece3.2405 28725376 PMC5513214

[7] de Vlaming VL, Bury RB (1970) Thermal selection in tadpoles of the tailed-frog, *Ascaphus truei*. J Herpetol 4: 179–189.

[8] Packard GC, Packard MJ (1988) The physiological ecology of reptilian eggs and embryos. Biol Rev 52: 71–105. 10.1111/j.1469-185x.1977.tb01346.x 319843

[9] Miller K, Packard GC (1977) An altitudinal cline in critical thermal maxima of chorus frogs (*Pseudacris triseriata*). Am Nat 111: 267–277.

[10] McCann S, Greenlees M, Newell D, Shine R (2014) Rapid acclimation to cold allows the cane toad to invade montane areas within its Australian range. Funct Ecol 28: 1166–1174.

[11] McCann S, Kosmala G, Greenlees M, Shine R (2018) Physiological plasticity in a successful invader: rapid acclimation to cold occurs only in cool-climate populations of cane toads (*Rhinella marina*). Conserv Physiol 6: cox072. doi: 10.1093/conphys/cox072 29399360 PMC5786208

[12] Brown HA (1969) The heat resistance of some anuran tadpoles (Hylidae and Pelobatidae). Copeia 1969: 138–147.

[13] Méndez MA, Correa-Solis M (2009) Divergence in morphometric and life history traits in two thermally contrasting Andean populations of *Rhinella spinulosa* (Anura: Bufonidae). J Therm Biol 34: 342–347.

[14] Brown HA (1975) Embryonic temperature adaptations of the pacific treefrog, *Hyla regilla*. Comp Biochem Physiol A 51: 863–873. doi: 10.1016/0300-9629(75)90067-5 237710

[15] Berven KA, Gill DE, Smith-Gill SJ (1979) Countergradient selection in the green frog, *Rana clamitans*. Evolution 33: 609–623. doi: 10.1111/j.1558-5646.1979.tb04714.x 28563934

[16] Prentis PJ, Wilson JRU, Dormontt EE, Richardson DM, Lowe AJ (2008) Adaptive evolution in invasive species. Trends Plant Sci 13: 288–294. doi: 10.1016/j.tplants.2008.03.004 18467157

[17] Davidson AM, Jennions M, Nicotra AB (2011) Do invasive species show higher phenotypic plasticity than native species and, if so, is it adaptive? A meta‐analysis. Ecol Lett 14: 419–431. doi: 10.1111/j.1461-0248.2011.01596.x 21314880

[18] Tepolt CK, Somero GN (2014) Master of all trades: thermal acclimation and adaptation of cardiac function in a broadly distributed marine invasive species, the European green crab, *Carcinus maenas*. J Exp Biol 217: 1129–1138. doi: 10.1242/jeb.093849 24671964

[19] Hill J, Thomas C, Blakeley D (1999) Evolution of flight morphology in a butterfly that has recently expanded its geographic range. Oecologia 121: 165–170. doi: 10.1007/s004420050918 28308556

[20] Lee CE (2002) Evolutionary genetics of invasive species. Trends Ecol Evol 17: 386–391.

[21] Cox GW (2004) Alien species and evolution: The evolutionary ecology of exotic plants, animals, microbes, and interacting native species. Washington, DC: Island Press.

[22] Simmons AD, Thomas CD (2004) Changes in dispersal during species’ range expansions. Am Nat 164: 378–395. doi: 10.1086/423430 15478092

[23] Benkman CW, Siepielski AM, Parchman TL (2008) The local introduction of strongly interacting species and the loss of geographic variation in species and species interactions. Mol Ecol 17: 395–404. doi: 10.1111/j.1365-294X.2007.03368.x 18173508

[24] Shine R (2010) The ecological impact of invasive cane toads (*Bufo marinus*) in Australia. Q Rev Biol 85: 253–291. doi: 10.1086/655116 20919631

[25] Jolly C, Shine R, Greenlees MJ (2015) The impact of invasive cane toads on native wildlife in southern Australia. Ecol Evol 5: 3879–3894. doi: 10.1002/ece3.1657 26445649 PMC4588653

[26] Vanbeurden EK, Grigg GC (1980) An Isolated and expanding population of the introduced toad *Bufo marinus* in New South Wales. Aust Wildl Res 7: 305–310.

[27] Greenlees MJ, Harris S, White AW, Shine R (2018) The establishment and eradication of an extra-limital population of invasive cane toads. Biol Invasions 20: 2077–2089.

[28] Greenlees M, Brown GP, Shine R (2020) Pest control by the public: impact of hand-collecting on the abundance and demography of cane toads (*Rhinella marina*) at their southern invasion front in Australia. Global Ecol Conserv 23: e01120.

[29] Macgregor LF, Greenlees M, de Bruyn M, Shine R (2021) An invasion in slow motion: the spread of invasive cane toads (*Rhinella marina*) into cooler climates in southern Australia. Biol Invasions. doi: 10.1007/s10530-021-02597-2

[30] Kearney M, et al (2008) Modelling species distributions without using species distributions: the cane toad in Australia under current and future climates. Ecography 31: 423–434.

[31] Phillips BL, Brown GP, Webb JK, Shine R (2006) Invasion and the evolution of speed in toads. Nature 439: 803. doi: 10.1038/439803a 16482148

[32] Hudson CM, Vidal-García M, Murray TG, Shine R (2020) The accelerating anuran: evolution of locomotor performance in cane toads (*Rhinella marina*, Bufonidae) at an invasion front. Proc R Soc B 287: 20201964. doi: 10.1098/rspb.2020.1964 33171090 PMC7735276

[33] Phillips B, Shine R (2006) Spatial and temporal variation in the morphology (and thus, predicted impact) of an invasive species in Australia. Ecography 29: 205–212.

[34] Phillips BL (2009) The evolution of growth rates on an expanding range edge. Biol Lett 5: 802–804. doi: 10.1098/rsbl.2009.0367 19605384 PMC2827979

[35] Stuart K, Shine R, Brown GP (2019) Proximate mechanisms underlying the rapid modification of phenotypic traits in cane toads (*Rhinella marina*) across their invasive range within Australia. Biol J Linn Soc 126: 68–79.

[36] Tingley R, Greenlees MJ, Shine R (2012) Hydric balance and locomotor performance of an anuran (*Rhinella marina*) invading the Australian arid zone. Oikos 121: 1959–1965.

[37] Kosmala G, Brown GP, Shine R, Christian K (2020) Skin resistance to water gain and loss has changed in cane toads (*Rhinella marina*) during their Australian invasion. Ecol Evol 10: 13071–13079. doi: 10.1002/ece3.6895 33304517 PMC7713918

[38] Kosmala G, Brown GP, Christian K, Hudson CM, Shine R (2018) The thermal dependency of locomotor performance evolves rapidly within an invasive species. Ecol Evol 8: 4403–4408. doi: 10.1002/ece3.3996 29760882 PMC5938468

[39] Tingley R, Shine R (2011) Desiccation risk drives the spatial ecology of an invasive anuran (*Rhinella marina*) in the Australian semi-desert. PLOS ONE 6: e25979. doi: 10.1371/journal.pone.0025979 22043300 PMC3197141

[40] Hagman M, Shine R (2006) Spawning site selection by feral cane toads (*Bufo marinus*) at an invasion front in tropical Australia. Austral Ecol 31: 551–558.

[41] Evans M, Yáber C, Hero J-M (1996) Factors influencing choice of breeding site by *Bufo marinus* in its natural habitat. Copeia 1996: 904–912.

[42] Volpe EP (1957) Embryonic temperature tolerance and rate of development in *Bufo valliceps*. Physiol Zool 30: 164–176.

[43] Capellán E, Nicieza AG (2007) Trade-offs across life stages: does predator–induced hatching plasticity reduce anuran post-metamorphic performance? Evol Ecol 21: 445–458.

[44] Cabrera-Guzmán E, Crossland MR, Brown GP, Shine R (2013) Larger body size at metamorphosis enhances survival, growth and performance of young cane toads (*Rhinella marina*). PLOS ONE 8: e70121. doi: 10.1371/journal.pone.0070121 23922930 PMC3726449

[45] García-Ramos G, Kirkpatrick M (1997) Genetic models of adaptation and gene flow in peripheral populations. Evolution 51: 21–28. doi: 10.1111/j.1558-5646.1997.tb02384.x 28568782

[46] White AW, Shine R (2009) The extra-limital spread of an invasive species via ‘stowaway’ dispersal: toad to nowhere? Anim Conserv 12: 38–45.

[47] Savolainen O, Pyhäjärvi T, Knürr T (2007) Gene flow and local adaptation in trees. Annu Rev Ecol Evol System 38: 595–619.

[48] Semeniuk M, Lemckert F, Shine R (2007) Breeding-site selection by cane toads (*Bufo marinus*) and native frogs in northern New South Wales, Australia. Wildl Res 34: 59–66.

[49] Gilchrist G, Lee C (2007) All stressed out and nowhere to go: does evolvability limit adaptation in invasive species? Genetica 129: 127–132. doi: 10.1007/s10709-006-9009-5 16924404

[50] Wijethunga U, Greenlees M, Shine R (2015) The acid test: pH tolerance of the eggs and larvae of the invasive cane toad (*Rhinella marina*) in southeastern Australia. Physiol Biochem Zool 88: 433–443. doi: 10.1086/681263 26052640

[51] Lever C (2001) The cane toad. The history and ecology of a successful colonist. Otley, West Yorkshire: Westbury Academic and Scientific Publishing.

[52] Kelehear C, Webb J, Shine R (2009) *Rhabdias pseudosphaerocephala* infection in *Bufo marinus*: lung nematodes reduce viability of metamorph cane toads. Parasitology 136: 919–927. doi: 10.1017/S0031182009006325 19523249

